# Short Tear Breakup Time Could Exacerbate the Progression of Presbyopia in Women

**DOI:** 10.1155/2022/8159669

**Published:** 2022-01-28

**Authors:** Masahiko Ayaki, Kazuno Negishi

**Affiliations:** ^1^Department of Ophthalmology, Keio University School of Medicine, Tokyo, Japan; ^2^Otake Clinic Moon View Eye Center, Kanagawa, Japan

## Abstract

**Purpose:**

The contributory factors and symptoms for presbyopia progression have not been fully determined. The purpose of the study was to compare presbyopia progression in subjects with short and normal tear breakup time and to explore the severity of common ocular symptoms associated with presbyopia progression.

**Method:**

We conducted a clinic-based, retrospective, cross-sectional study. Inclusion criteria were bilateral phakic patients aged 40–69 years with best-corrected distance visual acuity better than 20/30, and exclusion criteria were the use of glaucoma eye drops, any disease affecting vision, or history of ocular surgery. We measured the binocular near add power and compared the results using Kaplan-Meier analysis. Association between near add power and ocular symptoms was explored.

**Results:**

There were 1411 participants (mean age of 50.1 years). There were no significant differences in age, intraocular pressure, spherical equivalent, astigmatism, or anisometropia between the sexes. Kaplan-Meier analysis indicated that women with short tear breakup time reached the endpoint (near add power of +3.00 D) significantly earlier than those with normal tear breakup time (*P* = 0.043; Cox-Mantel test). Eye fatigue was most severe in the group with an add power of 1.25-2.00 D. Near add power was correlated with hyperopia, astigmatic errors, and anisometropia.

**Conclusions:**

This study suggests an exacerbation of presbyopia progression in women with short tear breakup time. Eye fatigue was most severe in those with an add power of 1.25-2.00 D.

## 1. Introduction

Presbyopia is a consequence of aging with the loss of accommodation, and its progression predominantly depends on progressive lens hardening and decreased ciliary muscle mobility [1]. Many middle-aged people may suffer presbyopia, and previous studies have described the impact of presbyopia involving disability, economic burden [2–7], and deterioration of functional visual acuity, quality of life, subjective happiness, and sleep [8–11]. A recent meta-analysis found the age-adjusted global prevalence of blindness has reduced over the past three decades. However, even with normal distance vision, near vision impairment by uncorrected presbyopia (called functional presbyopia) is increasing due to global population growth and aging [2]. This survey defined vision impairment from uncorrected presbyopia as presenting near vision <N6 or <N8 at 40 cm, where best-corrected distance visual acuity is ≥6/12. The burden would increase with the growing need for near vision in rapidly digitalized societies involving pre- and early-presbyopes. Awareness, impact, and progression of presbyopia may depend on various factors, including sex, work, and habit; however, this has not been fully determined [12–15].

Dry eye (DE) is a common age-related eye disease in those of presbyopic age and more prevalent in women than men [16]. DE is a multifactorial ocular surface disease characterized by unstable and/or deficient tear film accompanied by ocular surface epitheliopathy, inflammation, and neurosensory abnormalities [17]. DE has become more prevalent during the COVID-19 pandemic [18–20]. It may cause visual problems, including eye fatigue and photophobia. Visual function diminishes in DE [21] due to higher-order aberration [22], irregular astigmatism [22], decreased contrast sensitivity [23, 24], and functional visual acuity [25–27] due to corneal epitheliopathy and tear film instability between blinking. Visual function in DE can now be measured with new instruments including optical coherence tomography [28–30], wavefront sensor [31], and videokeratography [32]. DE-associated visual problems are a considerable issue in refractive and cataract surgery, and a diagnostic algorithm has been developed to manage visually significant DE before surgery [33]. Topical treatment was shown to improve visual function in short tear breakup time- (BUT-) type DE [34–36]. No study has investigated the relationship between DE and presbyopia to date, and we hypothesize that DE may have further impact on presbyopia in middle adulthood.

The aim of this study was to investigate the effect of DE-related parameters on near vision. We focused on BUT, which may affect vision, especially in middle- to older-aged DE patients. We achieved this by measuring the near add power (the minimal additional power required to achieve sufficient near acuity under full distance refractive correction) in outpatients, comparing the progression of presbyopia between these patients stratified by sex. The associations between near add power and DE-related symptoms and ocular parameters were also explored.

## 2. Methods

### 2.1. Study Design and Participants

This study was a clinic-based, retrospective, cross-sectional study involving healthy subjects attending the Tsukuba Central Hospital and Otake Clinic Moon View Eye Center. The Institutional Review Board and Ethics Committee of the Tsukuba Central Hospital (approved on 12 December 2014, permission number 141201) and Kanagawa Medical Association (approved on 12 November 2018, permission number krec2059006) approved the study, and it was carried out in accordance with the Declaration of Helsinki. The need for consent was waived by the Institutional Review Board. This study was a retrospective chart review since patient interview and ocular examinations for analysis were routinely performed in the participating eye clinics. The Institutional Review Board and Ethics Committee of Keio University School of Medicine approved this study (approval date 28 June 2021; approval number 20210080) to permit authorship for authors (KN and MA) who were appointed at the Keio University School of Medicine.

### 2.2. Inclusion and Exclusion Criteria

Participants aged 40 to 69 years with bilateral phakic eyes and best-corrected visual acuity above 20/30 were included. Individuals were excluded if they used antiglaucoma or anticataract eye drops or had vitreoretinal disease, any ocular surgery in the previous month, or acute ocular disease in the previous two weeks.

### 2.3. Ophthalmological Examinations

Board-certified ophthalmologists examined all patients and excluded subjects with major age-related eye diseases, including cataract, glaucoma, and macular diseases. Ophthalmological evaluation of participants consisted of best-corrected visual acuity (Vision Chart, SSC-370^R^, Nidek Co., Ltd., Gamagori, Japan), autorefractometry (Tonoref™ II, Nidek Co., Ltd., Aichi, Japan), slit-lamp biomicroscopy, funduscopy, and intraocular pressure measurements (Tonoref™ II, Nidek Co., Ltd., Aichi, Japan). Binocular near add power was measured by a blinded examiner at a distance of 30 cm using a Bankoku near-acuity chart (Handaya Inc., Tokyo, Japan) or an automatic optometry system (AOS-700^R^; Nidek Co., Ltd., Aichi, Japan) [11, 37]. After determining the patient's distance refractive correction, the minimal additional power required to achieve near acuity above 20/25 at 30 cm was measured in 0.25 D increments and was recorded as near add power. DE-related examinations consisted of BUT, corneal staining test, Schirmer test, and tear strip meniscometry. They were performed according to standard procedures [38]. BUT was measured using wet fluorescein filter paper (Ayumi Pharmaceutical, Tokyo, Japan) applied at the lower lid margin. The fluorescein strip was wet with saline, and the excess was flicked off. BUT was defined as the time interval between the third blink and the appearance of the first dark spot in the cornea. This was calculated from the mean of three measurements. A corneal staining score was determined to grade corneal epitheliopathy, graded at 0–2 for severity and area. Schirmer's test was performed without topical anaesthesia. Strips of filter paper (Whatman No. 41; Showa Yakuhin Kako, Tokyo, Japan) were placed for five minutes at the outer third of the temporal lower conjunctival fornix. The strips were then removed, and the length of the filter paper wetted by the spontaneous blinks was recorded (mm). Tear strip meniscometry was performed by inserting the strip (SMTube, Echo Electricity Co., Ltd., Fukushima, Japan) for five seconds into the lateral side of the lower lid tear meniscus without touching the ocular surface to measure aqueous availability [39]. Examination rooms were kept at 21-24 degrees centigrade and 40-60% humidity according to the recommendation of the Japanese Ministry of Health and Labor.

### 2.4. Patient Interviews for DE-Related Symptoms

Patients were asked questions to determine the presence or absence of six common DE-related symptoms: dryness, irritation, pain, eye fatigue, blurring, and photophobia. These questions were selected from items on the Dry Eye-Related Quality-of-Life Score (DEQS) questionnaire [40] and were based on the six most prevalent symptoms of DE in patients who had visited the Dry Eye Clinic in the Department of Ophthalmology at Keio University Hospital in 2014.

### 2.5. Statistical Analysis

The sample size was calculated with a 0.05 margin of error and 95% confidence interval. Effect size was derived from a measured value in the current study. An effect size of 0.505 was identified in near add power with an appropriate total sample size for comparison of men and women being 34. Patient demographics and ophthalmological parameters were compared between sexes using the *t*-test and chi-squared test, as appropriate. Near add power with an endpoint of +3.00 D was compared between short BUT (≤2 mm) and normal BUT (>2 mm) patients using Kaplan-Meier analysis and the Cox-Mantel test. Regression analyses were performed to explore the correlation between near add power and ophthalmological parameters. To assess the association between near add power and common ocular symptoms, near add power was classified as low (+0.25 to +1.00 D; *n* = 445), moderate (+1.25 to +2.00 D; *n* = 471), or high (+2.25 to +3.00 D; *n* = 446). Data are presented as the mean ± standard deviation (SD) or as percentages where appropriate. All analyses were performed using StatFlex (Atech, Osaka, Japan), with *P* < 0.05 considered significant.

## 3. Results

**Figure 1:**
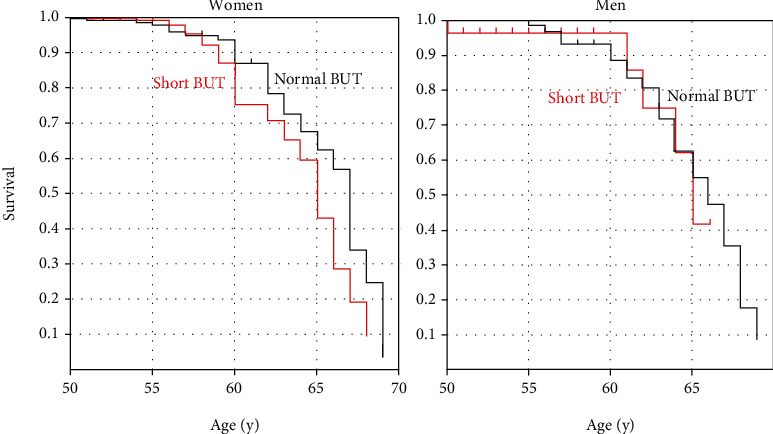
Kaplan-Meier plot of (a) women and (b) men showing the age at which individuals in the short (red line) and normal (black line) BUT groups reached the near add power endpoint of +3.00 D. The short BUT group reached the endpoint of +3.00 D significantly earlier than the normal BUT group (*P* = 0.043; Cox-Mantel test) in women, but this was not observed in men (*P* = 0.759). BUT: tear breakup time.

There were 1411 participants (1063 women and 348 men, mean age 50.6 ± 7.6 y). There were no significant differences in age, intraocular pressure, spherical equivalent, astigmatic errors, anisometropia, near add power, and Schirmer test value between the sexes, whilst BUT, corneal staining score, and tear strip meniscometry were worse in women (Table 1). BUT was measured in 1030 patients, and the number of normal BUT and short BUT was 480 (63.8%) and 272 (36.2%), respectively, in women, and 230 (82.7%) and 48 (17.3%), respectively, in men with a significant sex difference (*P* < 0.001, chi-squared test). All symptoms were more prevalent in women than in men (Table 2). A Kaplan-Meier plot revealed the near add power of the short BUT group reached an endpoint of +3.00 D, which was significantly earlier than the normal BUT group in women (*P* = 0.043, Cox-Mantel test), but not in men (*P* = 0.759; Figure 1). Kaplan-Meier analysis showed that there was no difference between women and men with normal BUT (*P* = 0.790) and with short BUT (*P* = 0.723).

**Figure 2:**
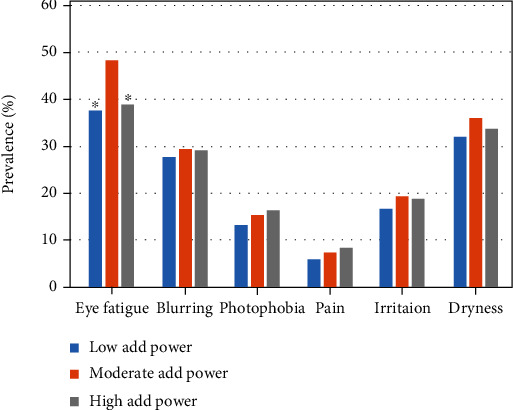
Prevalence of symptoms and near add power. Near add power was classified as low (+0.25 to +1.00 D), moderate (+1.25 to +2.00 D), and high (+2.25 to +3.00 D). A significant difference was observed in the prevalence of eye fatigue (vs. moderate add power group, chi-squared test). ^∗^*P* < 0.001.

Regression analysis revealed near add power correlated with spherical equivalent (*β* = −0.040, *P* < 0.001), astigmatic errors (*β* = 0.059, *P* < 0.001), anisometropia (*β* = 0.041, *P* = 0.008), and corneal staining score (*β* = 0032, *P* = 0.045), adjusted for age and sex (Table 3). The association between progression of near add power and common ocular symptoms and ocular parameters was then assessed. A comparison of the three groups classified by near add power found spherical equivalent, astigmatic error, Schirmer test value, and tear strip meniscometry were significantly different among the groups (Table 4). Specifically, spherical equivalent was hyperopic and astigmatic errors were greater in the high add power group. In contrast, symptoms did not differ among groups, except that a significant difference was observed in the prevalence of eye fatigue between the moderate add power group compared with the other two groups (*P* < 0.001, chi-squared test; Figure 2).

## 4. Discussion

Our results suggest an exacerbation of presbyopia progression in women with short BUT, with whom decreased image quality with short BUT may have a greater impact compared with men. Mai et al. analyzed the Taiwan National Health Insurance Research Database and found a significant association between presbyopia and DE even after matching age/gender and comorbidity conditions [41]. Kaido et al. found accommodative microfluctuation in DE patients and hypothesized that eye fatigue symptoms may develop from ciliary muscle spasms caused by image blurring due to tear instability in DE [42]. We hypothesize that accommodation may be diminished by ciliary muscle spasms, and progression of presbyopia may be faster in short BUT-type DE. Whilst presbyopia affects both sexes equally, with the amplitude of accommodation being the same in men and women of the same age, previous study suggested women suffered from it less severely [11]. The present results suggest the burden of presbyopia is not directly associated with the additional power requirements for near vision. Rather, it may depend on refraction, preferred distance correction, and preferred viewing distances. Women may tend to use more near vision than men and, accordingly, adapt presbyopia to their lifestyle with preferred near and distant correction [11] despite presbyopia progression with DE as indicated in this study. Short BUT type is a typical DE symptom [17], and it may be a serious health problem in middle adulthood. Topical medications of mucin secretagogue were proven effective in improving visual function in short BUT-type DE [34–36], and adequate management of DE may improve visual function and may be expected to suppress progression of presbyopia in short BUT cases. In contrast, short BUT was not as prevalent in men and, as such, further investigation with larger cohorts is required to confirm any associations between presbyopia progression and BUT in men.

The prevalence of eye fatigue was most severe in the group with a near add power of 1.25 to 2.00 D; however, this finding needs to be confirmed in additional analyses that adjust for sex and age. The low add power group may tolerate uncorrected presbyopia by adjusting distance at near work, using undercorrected spectacles or contact lens for myopic errors. The high add power group may use near corrective devices and therefore not suffer focusing difficulties for near. In contrast, the moderate add power group may be in a transition period for starting to use corrective devices for near vision as a previous study indicated presbyopic burden started at around 47 years of age [11], which is between the low and middle add power groups in the present study.

Regression analysis revealed hyperopia, astigmatic errors, and anisometropia weakly correlated with near add power, which is comparable with previous investigations [14, 15, 43]. Anisometropia may disrupt binocular vision and result in impaired near vision [15]. Based on the present results, physicians and patients should be aware that DE may worsen presbyopia in women, in addition to hyperopia, astigmatic errors, and anisometropia. Appropriate correction of near addition, refractive errors, and anisometropia may help relieve the burden of presbyopia. Presbyopia and DE may worsen the quality of life in middle and older ages [11, 43–46], and both common disorders should be recognized in eye care practice. Future research in this area should assess the effect of DE management, including medical and surgical measures, on presbyopia progression.

Despite the promising findings, this study has some limitations. In this study, corneal and lacrimal examinations were performed by a board-certified ophthalmologist (MA) and visual acuity and near add power were examined by five certified orthoptists with national licensure. The presence of an intra- and interindividual error rate should have been assessed [47, 48]; however, this has not been done and this was a considerable limitation. The number of men studied was small, and larger prospective studies are required to provide more conclusive results about the progression of presbyopia in male DE patients. Characteristics including systemic comorbidities and other possible factors should also be investigated as potential contributors to presbyopia progression. Measuring pupillary diameter, aberrations, anterior chamber depth, lens thickness, lens capsule curvature, and axial length could also provide knowledge about the structural changes that take place in the globe during presbyopia progression in short and normal BUT. Objective accommodation measures might further enhance our understanding of the effects of DE and BUT on accommodation.

## Figures and Tables

**Table 1:** Patient demographics and ophthalmological parameters.

	Total	Women	Men	*P* value^∗^
Number of cases	1411	1063	348	
Age (y)	50.6 ± 7.6	50.6 ± 7.3	50.6 ± 7.5	0.986
Spherical equivalent (D)	−3.41 ± 3.15	−3.43 ± 3.14	−3.35 ± 3.21	0.654
Astigmatic errors (D)	0.58 ± 0.64	0.57 ± 0.62	0.60 ± 0.71	0.444
Anisometropia (D)	0.58 ± 0.78	0.56 ± 0.70	0.65 ± 0.99	0.100
Near add power (D)	1.56 ± 0.99	1.56 ± 0.99	1.53 ± 0.99	0.713
BUT (s)	3.93 ± 2.06	3.50 ± 2.06	5.45 ± 1.17	**<0.001**
(% of BUT < 3 mm)	(31.1)	(36.2)	(17.3)	**(<0.001)**
Corneal staining score	0.27 ± 0.56	0.32 ± 0.60	0.10 ± 0.33	**<0.001**
Schirmer test value (mm)	11.4 ± 8.8	11.3 ± 8.8	11.9 ± 9.3	0.903
Tear strip meniscometry (mm)	1.67 ± 2.48	1.39 ± 2.12	3.00 ± 3.53	**0.014**

^∗^Women vs. men, unpaired *t*-test. BUT: tear breakup time.

**Table 2:** Prevalence of symptoms in women and men.

Symptom	Total (%)	Women (%)	Men (%)	*P* value^∗^
Dryness	34.0	39.1	16.1	**<0.001**
Irritation	18.1	21.0	7.3	**<0.001**
Pain	7.1	7.9	4.0	**0.036**
Fatigue	41.7	44.1	32.7	**0.001**
Blurring	28.4	30.9	19.0	**<0.001**
Photophobia	15.0	16.3	10.1	**0.014**

^∗^Women vs. men, chi-squared test.

**Table 3:** Correlation between near add power and parameters.

Parameters	Linear regression^∗^		Adjusted for age and sex^∗^	
	*β*	*P* value	*β*	*P* value
Age	0.8153	**<0.001**	0.797	**<0.001**
Sex	0.009	0.737	0.001	0.927
Spherical equivalent	0.115	**<0.001**	−0.040	**0.010**
Astigmatic errors	0.164	**<0.001**	0.059	**<0.001**
Anisometropia	0.035	0.194	0.041	**0.008**
Tear breakup time	−0.061	**0.047**	−0.012	0.527
Corneal staining score	0.015	0.572	0.032	**0.045**
Schirmer test value	−0.203	**0.009**	−0.077	0.477
Tear strip meniscometry	−0.068	0.505	−0.010	0.900

^∗^Standardized partial regression coefficient.

**Table 4:** Demographics of presbyopia groups.

	Low add power	Moderate add power	High add power	*P* value^∗^
Number of cases	445	471	446	
% of men	24.5	27.2	24.9	0.837
Age (y)	44.0 ± 5.5	49.6 ± 5.5	58.4 ± 7.4	**<0.001**
Spherical equivalent (D)	−3.66 ± 3.01	−3.72 ± 3.54	−2.85 ± 3.15	**<0.001**
Astigmatic errors (D)	0.47 ± 0.62	0.58 ± 0.66	0.69 ± 0.64	**<0.001**
Anisometropia (D)	0.55 ± 0.70	0.56 ± 0.99	0.63 ± 0.78	0.141
Near add power (D)	0.34 ± 0.82	1.68 ± 0.22	2.63 ± 0.98	**<0.001**
Intraocular pressure (mmHg)	14.6 ± 2.9	14.5 ± 2.8	14.2 ± 2.9	0.075
Tear breakup time (s)	4.12 ± 2.08	3.94 ± 1.97	3.77 ± 2.06	0.084
Corneal staining score	0.26 ± 0.57	0.27 ± 0.50	0.28 ± 0.56	0.839
Schirmer test value (mm)	14.41 ± 9.36	12.22 ± 6.90	9.44 ± 8.89	**0.048**
Tear strip meniscometry (mm)	3.10 ± 2.49	1.48 ± 2.49	1.53 ± 2.48	**0.010**

^∗^Kruskal-Wallis test, except for % of men analyzed with Mann–Whitney *U* test.

## Data Availability

The corresponding author has all data and could disclose upon appropriate request.

## Abbreviations

DE: Dry eye
BUT: Tear breakup time.

## References

[1] Duane A. (1922). Studies in monocular and binocular accommodation with their clinical applications. *Transactions of the American Ophthalmological Society*.

[2] GBD 2019 Blindness and Vision Impairment Collaborators (2021). Trends in prevalence of blindness and distance and near vision impairment over 30 years: an analysis for the Global Burden of Disease Study. *The Lancet Global Health*.

[3] Holden B. A., Fricke T. R., Ho S. M. (2008). Global vision impairment due to uncorrected presbyopia. *Archives of Ophthalmology*.

[4] Frick K. D., Joy S. M., Wilson D. A., Naidoo K. S., Holden B. A. (2015). The global burden of potential productivity loss from uncorrected presbyopia. *Ophthalmology*.

[5] Fricke T. R., Tahhan N., Resnikoff S. (2018). Global prevalence of presbyopia and vision impairment from uncorrected presbyopia: systematic review, meta-analysis, and modelling. *Ophthalmology*.

[6] Sharma G., Chiva-Razavi S., Viriato D. (2020). Patient-reported outcome measures in presbyopia: a literature review. *BMJ Open Ophthalmology*.

[7] Berdahl J., Bala C., Dhariwal M., Lemp-Hull J., Thakker D., Jawla S. (2020). Patient and economic burden of presbyopia: a systematic literature review. *Clinical Ophthalmology*.

[8] Katada Y., Negishi K., Watanabe K. (2016). Functional visual acuity of early presbyopia. *PLoS One*.

[9] Muhammad N., Alhassan M. B., Umar M. M. (2015). Visual function and vision-related quality of life in presbyopic adult population of northwestern Nigeria. *Nigerian Medical Journal*.

[10] Goertz A. D., Stewart W. C., Burns W. R., Stewart J. A., Nelson L. A. (2014). Review of the impact of presbyopia on quality of life in the developing and developed world. *Acta Ophthalmologica*.

[11] Negishi K., Ayaki M., Kawashima M., Tsubota K. (2021). Sleep and subjective happiness between the ages 40 and 59 in relation to presbyopia and dry eye. *PLoS One*.

[12] Hickenbotham A., Roorda A., Steinmaus C., Glasser A. (2012). Meta-analysis of sex differences in presbyopia. *Investigative Ophthalmology & Visual Science*.

[13] Kubota M., Kubota S., Kobashi H., Ayaki M., Negishi K., Tsubota K. (2020). Difference in pupillary diameter as an important factor for evaluating amplitude of accommodation: a prospective observational study. *Journal of Clinical Medicine*.

[14] Rabbetts R. B. (1998). *Accommodation and Near Vision. The Inadequate- Stimulus Myopias*.

[15] Reindel W., Zhang L. N., Chinn J., Rah M. (2018). Evaluation of binocular function among pre- and early-presbyopes with asthenopia. *Clinical Optometry*.

[16] Choi H. R., Kim N. H., Lee J. M. (2021). Risk factors influencing the occurrence and severity of symptomatic dry eye syndrome: a cross-sectional study. *Ophthalmic Epidemiology*.

[17] Yokoi N., Uchino M., Uchino Y. (2015). Importance of tear film instability in dry eye disease in office workers using visual display terminals: the Osaka study. *American Journal of Ophthalmology*.

[18] Napoli P. E., Nioi M., Fossarello M. (2021). The "Quarantine Dry Eye": The Lockdown for Coronavirus Disease 2019 and Its Implications for Ocular Surface Health. *Risk Management and Healthcare Policy*.

[19] Arriola-Villalobos P., Burgos-Blasco B., Vidal-Villegas B. (2021). Effect of face mask on tear film stability in eyes with moderate-to-severe dry eye disease. *Cornea*.

[20] Koh S., Rhee M. K. (2021). COVID-19 and dry eye. *Eye & Contact Lens*.

[21] Rieger G. (1992). The importance of the precorneal tear film for the quality of optical imaging. *The British Journal of Ophthalmology*.

[22] Koh S. (2018). Irregular astigmatism and higher-order aberrations in eyes with dry eye disease. *Investigative Ophthalmology & Visual Science*.

[23] Koh S., Maeda N., Ikeda C. (2017). The effect of ocular surface regularity on contrast sensitivity and straylight in dry eye. *Investigative Ophthalmology & Visual Science*.

[24] Szczotka-Flynn L. B., Maguire M. G., Ying G. S. (2019). Impact of dry eye on visual acuity and contrast sensitivity: dry eye assessment and management study. *Optometry and Vision Science*.

[25] Kaido M. (2018). Functional visual acuity. *Investigative Ophthalmology & Visual Science*.

[26] Kaido M., Ishida R., Dogru M., Tsubota K. (2011). The relation of functional visual acuity measurement methodology to tear functions and ocular surface status. *Japanese Journal of Ophthalmology*.

[27] Goto E., Yagi Y., Kaido M., Matsumoto Y., Konomi K., Tsubota K. (2003). Improved functional visual acuity after punctal occlusion in dry eye patients. *American Journal of Ophthalmology*.

[28] Napoli P. E., Nioi M., d’Aloja E., Fossarello M. (2019). The Bull's Eye Pattern of the Tear Film in Humans during Visual Fixation on En-Face Optical Coherence Tomography. *Scientific Reports*.

[29] Napoli P. E., Nioi M., Mangoni L. (2020). Fourier-domain OCT imaging of the ocular surface and tear film dynamics: a review of the state of the art and an integrative model of the tear behavior during the inter-blink period and visual fixation. *Journal of Clinical Medicine*.

[30] Lee K. B., Koh K. M., Kwon Y. A., Song S. W., Kim B. Y., Chung J. L. (2017). Changes in tear volume after 3% diquafosol treatment in patients with dry eye syndrome: an anterior segment spectral-domain optical coherence tomography study. *Korean Journal of Ophthalmology*.

[31] Koh S., Maeda N., Kuroda T. (2002). Effect of tear film break-up on higher-order aberrations measured with wavefront sensor. *American Journal of Ophthalmology*.

[32] Goto T., Zheng X., Klyce S. D. (2003). A new method for tear film stability analysis using videokeratography. *American Journal of Ophthalmology*.

[33] Starr C. E., Gupta P. K., Farid M. (2019). An algorithm for the preoperative diagnosis and treatment of ocular surface disorders. *Journal of Cataract and Refractive Surgery*.

[34] Koh S., Maeda N., Ikeda C. (2014). Effect of diquafosol ophthalmic solution on the optical quality of the eyes in patients with aqueous-deficient dry eye. *Acta Ophthalmologica*.

[35] Kaido M., Uchino M., Kojima T., Dogru M., Tsubota K. (2013). Effects of diquafosol tetrasodium administration on visual function in short break-up time dry eye. *Journal of Ocular Pharmacology and Therapeutics*.

[36] Koh S., Inoue Y., Sugmimoto T., Maeda N., Nishida K. (2013). Effect of rebamipide ophthalmic suspension on optical quality in the short break-up time type of dry eye. *Cornea*.

[37] Ayaki M., Tsuneyoshi Y., Yuki K., Tsubota K., Negishi K. (2019). Latanoprost could exacerbate the progression of presbyopia. *PLoS One*.

[38] Negishi K., Ayaki M., Uchino M., Takei K., Tsubota K. (2020). Strip meniscometry correlates with ocular surface tests and symptoms. *Translational Vision Science & Technology*.

[39] Ayaki M., Miyasaka K., Otake H., O'hira A., Arai M., Sakai T. (2021). Tear strip meniscometry and its clinical application: 2000+ large case series. *Investigative Ophthalmology & Visual Science*.

[40] Sakane Y., Yamaguchi M., Yokoi N. (2013). Development and validation of the dry eye-related quality-of-life score questionnaire. *JAMA Ophthalmology*.

[41] Mai E. L. C., Lin C. C., Lian I., Liao R., Chen M., Chang C. (2019). Population-based study on the epidemiology of dry eye disease and its association with presbyopia and other risk factors. *International Ophthalmology*.

[42] Kaido M., Kawashima M., Shigeno Y., Tsubota K., Yamada Y. (2017). Relation of accommodative microfluctuation with dry eye symptoms in short tear break-up time dry eye. *PLoS One*.

[43] Negishi K., Toda I., Ayaki M., Torii H., Tsubota K. (2020). Subjective happiness and satisfaction in postoperative anisometropic patients after refractive surgery for myopia. *Journal of Clinical Medicine*.

[44] Jaiswal S., Asper L., Long J., Lee A., Harrison K., Golebiowski B. (2019). Ocular and visual discomfort associated with smartphones, tablets and computers: what we do and do not know. *Clinical & Experimental Optometry*.

[45] Coles-Brennan C., Sulley A., Young G. (2019). Management of digital eye strain. *Clinical & Experimental Optometry*.

[46] Morthen M. K., Magno M. S., Utheim T. P., Snieder H., Hammond C. J., Vehof J. (2021). The physical and mental burden of dry eye disease: a large population-based study investigating the relationship with health-related quality of life and its determinants. *The Ocular Surface*.

[47] Napoli P. E., Nioi M., Gabiati L. (2020). Repeatability and reproducibility of post-mortem central corneal thickness measurements using a portable optical coherence tomography system in humans: a prospective multicenter study. *Scientific Reports*.

[48] Napoli P. E., Nioi M., d’Aloja E., Fossarello M. (2016). Post-mortem corneal thickness measurements with a portable optical coherence tomography system: a reliability study. *Scientific Reports*.

